# Diabetes outbreak during COVID19 lock-down in a prediabetic patient with cystic fibrosis long treated with glargine

**DOI:** 10.1186/s13052-021-01076-7

**Published:** 2021-06-02

**Authors:** Francesco Maria Rosanio, Enza Mozzillo, Chiara Cimbalo, Alberto Casertano, Angela Sepe, Valeria Raia, Adriana Franzese, Antonella Tosco

**Affiliations:** 1 Regional Center of Pediatric Diabetes, Department of Translational Medical Science, Section of Pediatrics, Federico II University of Naples, Naples, Italy; 2 Regional Center of Cystic Fibrosis, Department of Translational Medical Science, Section of Pediatrics, Federico II University of Naples, Naples, Italy

**Keywords:** Cystic fibrosis related diabetes, Prediabetes, Glucose derangements, Insulin, Glargine, Oral glucose tolerance test, COVID19, Lockdown

## Abstract

**Background:**

Cystic Fibrosis Related Diabetes (CFRD) is a frequent comorbidity of patients with Cystic Fibrosis (CF). A worsening of clinical conditions appears before CFRD. It has been demonstrated a decline in pulmonary function and nutritional status also in patients with prediabetes. Few trials show that insulin may be beneficial in prediabetic CF patients, to date guidelines do not recommend for this condition.

**Case presentation:**

We report a case of a patient treated with insulin glargine at 13 years, due to glycemic intolerance, and with Lumacaftor/Ivacaftor at 15 years. A reduction of pulmonary exacerbations was observed after glargine therapy, also confirmed after the starting of Lumacaftor/ Ivacaftor in this patient. Pulmonary function improved only after the first year of glargine therapy, then a deterioration appeared due to the natural history of CF lung damage. During the COVID-19 lockdown, poor adherence to care contributed to diabetes mellitus onset needing high insulin requirements. After two weeks the patient returned to prediabetic condition and his previous dose of glargine.

**Conclusions:**

our case highlights firstly that insulin glargine has contributed to preserve him from further clinical worsening due to prediabetes in the years before pandemic, secondly the negative impact of COVID-19 lockdown on the clinical course of a chronic disease as CF.

## Background

Cystic fibrosis (CF) is the most common autosomal recessive disease in Caucasians, with a worldwide prevalence of 1 in 2500 live births [1]. Patients with CF may develop numerous disease-related comorbidities, the most common is diabetes mellitus which manifests after a progressive reduction in glucose tolerance [2]. The pathophysiology of CF-related diabetes (CFRD) is extremely complex. As in type 1 diabetes mellitus (T1DM), progressive loss of pancreatic islet cells occurs in CFRD; however, unlike T1DM, beta cell autoimmunity markers are negative and the onset of diabetic ketoacidosis (DKA) is extremely rare since there is a minimal insulin production. As in type 2 diabetes mellitus (T2DM), insulin resistance occurs, linked to frequent infective pulmonary exacerbations or use of glucocorticoid agents. Glucose homeostasis is further disrupted by the requirement for high caloric intake, gut abnormalities and liver disease resulting in progressive insulin deficiency [3]. Since CFRD is often clinically silent, and glycosylated haemoglobin (HbA1c) is not considered a diagnostic tool, due to its small reliability with the glucose impairment, routine screening by oral glucose tolerance test (OGTT) is recommended. The OGTT is the only accepted screening test, starting from ten years of life and performed in stable baseline health conditions. Some authors suggested to extend OGTT test as screening of early glucose derangements under 10 years of age also [4, 5]. According to the American Diabetes Association (ADA) [6] and the ISPAD guidelines [7], diagnosis of diabetes can be made, during acute illnesses, when fasting plasma glucose (FPG) levels ≥126 mg/dL (≥ 7.0 mmol/L) or plasma glucose ≥200 mg/dL (≥ 11.1 mmol/L) persist for more than 48 h. During a period of stable baseline health, if FPG level is ≥126 mg/dL (≥7.0 mmol/L) or ≥ 200 mg/dL (≥11.1 mmol/L) at time (T) 120 of the OGTT; or if random blood glucose is > 200 mg / dL (> 11.1 mmol / L) on 2 or more occasions with symptoms, diagnosis of CFRD-fasting hyperglycemia plus (CFRD-FH+) and less (CFRD-FH-) can be respectively performed. Normal glucose tolerance (NGT) is defined by fasting blood glucose < 100 mg/dL (< 5.5 mmol/ L) or < 140 mg/dL (< 7.7 mmol/ L) at T120 of OGTT. The OGTT may reveal other prediabetic glucose alterations: impaired glucose tolerance (IGT), if blood glucose > 140 mg/dL (> 7.7 mmol/l) and < 200 mg/dL (< 11.1 mmol/l) at T120 and Indeterminate Glucose Tolerance (INDET) in NGT patients with one or more glucose values ≥200 mg/dL (≥11.1 mmol/L) at T30, T60 and/or T90 during OGTT [7]. Children with IGT or INDET compared to those with NGT show increased risk to develop CFRD [8, 9]. CFRD can also occur early in childhood [10] with increased prevalence as patients get older [2]. An insidious decline in clinical status of patients with CF showing early glucose alterations has been described in the years before the diagnosis of CFRD [11–14]. In children the decline in pulmonary function and nutritional parameters, correlated with insulin insufficiency, appear long before glucose levels are high enough to diagnose CFRD [15, 16] as in adults where prediabetes has a negative impact on the number of respiratory exacerbations, on the lung function and on the nutritional status even 2–4 years before CFRD diagnosis [17–19]. In IGT patients the drop in forced expiratory rate in the first second (FEV1) after four years of follow-up is higher as compared to NGT [11]. Insulin secretion in IGT is significantly lower than that of NGT patients, and the FEV1 positively correlates with beta cell function, as assessed by insulin resistance index HOMA% B [20]. The condition of insulin-deficiency determines an increase in protein catabolism [21]. Moreover, a more active inflammatory state (higher fibrinogen level) has been demonstrated in patients with IGT [20], therefore this may contribute to augment the pulmonary exacerbations. According to ISPAD guidelines [7] insulin therapy is the only recommended medical treatment for CFRD and evidence that insulin treatment may have an advantage on clinical outcomes of CF children showing early glucose derangements is scarce. In our previous report we showed that insulin treatment could represent an important strategy to improve lung function and BMI z-score, and to reduce pulmonary exacerbations in patients with CF showing prediabetic glucose alterations [14, 22]. We present a case of an adolescent with CFRD onset during the COVID-19 pandemic lockdown, but already treated with glargine basal insulin due to a prediabetic condition diagnosed four years earlier.

## Case presentation

S. is a 17 year old white male with CF (genotype delta F508 homozygous) associated to pancreatic insufficiency diagnosed at one month of life in therapy with lumacaftor/ivacaftor since he was 14 years. He had his first OGTT screening at 10 years of age. When he was 13-year old, diagnosis of IGT was made according to OGTT, 2-h glucose value was 142 mg/dL (7.9 mmol/L). Laboratory investigations showed that diabetes autoantibodies were negative while there had been an increase of HbA1C plasma value ranged from 5.7 to 6.5% (38 to 47.6 mmol/L). Since the patient in the last year had shown an impairment of the clinical conditions including decrease in percent predicted FEV1 (ppFEV1%) and BMI z-score, and increase in the number of lung infections, we decided to start off label use of insulin. After signing informed consent, basal insulin therapy with glargine at 0.1 units/Kg/day was prescribed and administered. After twelve months of glargine therapy, pulmonary exacerbations requiring intravenous antibiotic therapy were reduced from 4 to 2 exacerbations per year with a stabilization of FEV1% and BMI z-score. At the age of 15 years he started modulators therapy with the association of Lumacaftor/Ivacaftor [23]. For 2 years he had no need for intravenous antibiotic therapy with stable FEV1% and a slight increase of BMI z-score. At the age of 17 years, during COVID-19 pandemic lockdown, S. presented recurrent headache, abdominal pain and weight loss of 1.5 kg (BMI z-score − 2.4), polyuria and polydipsia. He was admitted to our Cystic Fibrosis Center in the suspicion of a pulmonary exacerbation. Physical examination showed dehydration and dystrophy, while chest auscultation showed crepitus and rales in the mid-apical lung fields bilaterally and clubbing, with oxygen saturation 96–97% in ambient air. The sudden loss of his father one month earlier, due to an acute myocardial infarction, was reported by the mother, who also referred a reduced adherence to all care of the patient, an excessive consumption of high-calorie foods associated with physical inactivity due to the lockdown, and a state of agitation. Laboratory investigations revealed glycemia 380 mg/dL (21.1 mmol/L), no chetoacidosis (pH 7.35, bicarbonate 23 mEq/L, beta-hydroxybutyrate 1 mmol/L), C-peptide 0.2 ng/mL, HbA1c 12.1% (108.5 mmol/L). The protein C reactive was 32.9 mg/l (normal range < 5) with blood count within normal range. Type 1 diabetes autoantibodies were retested and resulted negative. Nasopharyngeal swab and serological tests for SARS-CoV-2 were negative. Intravenous antibiotic therapy with amikacin and meropenem was started with normalization of inflammation markers. His insulin dose before lockdown was 5 units/day (0.10 units/kg/day). Insulin lispro was added to meals (with a carbohydrate ratio of 1 unit per 30 g carbohydrates, a sensitivity of 1 unit per 90 mg/dL) in addition to the increasing of insulin glargine for a total insulin requirement of 17 units/day (0.35 units/kg/day). A glucose monitoring system was started using an intermittent scanning sensor. After about 2 weeks, there was an improvement in glycemic control, ultrafast insulin was discontinued and S. returned to a pre-lockdown insulin dose. During hospitalization the main microvascular complications (i.e., microalbuminuria and retinopathy) were screened and resulted negative. At discharge, a new nasopharyngeal swab test for SARS-CoV-2 was repeated and resulted negative. To date, the insulin dose of S. has remained 0.1 units/kg/day (insulin glargine only), his BMI z-score has improved with a slight increase in FEV1%. Patient data before and after glargine and modulators therapy are presented in Table 1.

**Table 1:** Patient data before and after Glargine therapy

	Age (years)	Lumacaftor Ivacaftor	BMI z-score	FEV1 (%)	Annual number of pulmonary exacerbations	Annual number of pulmonary exacerbations treated by intravenous antibiotics
**2016 (before Glargine therapy)**	13.1	NO	−1.58	90.4%	7	4
**2017 (1 year of Glargine therapy)**	14.5	NO	−1.54	91%	4	2
**2018 (2 years of Glargine therapy)**	15.7	YES	−1.47	75%	2	0
**2019 (3 years of Glargine therapy)**	16.3	YES	−1.78	74%	3	0
**2020 (COVID-19 pandemic lockdown)**	17.0	YES	−2.47	68%	4	0
**2020 (November)**	17.6	YES	−1.58	71%	4	0

## Discussion and conclusions

Diabetes has a major negative impact on the clinical outcome of patients with CF. Although to a lesser extent, this has also been demonstrated in patients who show prediabetic alterations and who are at a high risk of developing diabetes. Identification of prediabetic CF patients may provide for improving or delaying clinical impairment [14]. According to ISPAD guidelines [7], there are no consistent recommendations to determine whether insulin treatment should also be used for all INDET and IGT patients, as well as in the previous ISPAD 2014 guidelines [24]. Through its promoting action on protein synthesis, insulin is known to improve nutritional and metabolic outcomes, and consequently lung function, even in patients with mild glycemic impairment. The IGT status indicates an insulin deficiency which leads to protein catabolism with a consequent negative impact on respiratory function by reducing diaphragm and intercostal muscle mass and strength. Early treatment with insulin could prevent this excessive catabolism [25]. Although there was no clear indication to start insulin therapy in S., who resulted IGT at the OGTT carried out in 2016, we decided to treat him with insulin, considering the severity of his underlying condition. Before starting insulin therapy S. showed frequent pulmonary flare-ups and difficulty in gaining and maintaining weight which required numerous hospitalizations for intravenous antibiotic therapy. After the initiation of insulin therapy we reported an important reduction of pulmonary exacerbations, probably explained by positive effect of glargine on anabolic metabolism and respiratory muscles strength. This positive trend was also confirmed after the starting of modulator therapy (Lumacaftor/ Ivacaftor). Regarding pulmonary function, S. showed an encouraging increasing of FEV1 only in the first year of insulin therapy, then an unrelenting deterioration appeared due to the natural history of CF lung damage, despite the Lumacaftor/Ivacaftor therapy. Respect to the nutritional status a noticeable impairment in BMI z score occurred when overt diabetes appeared. The latter was probably secondary to the poor adherence to therapy, the loss of the father, and the physical inactivity due to the COVID-19 lockdown. During lockdown S lifestyle dramatically changed determining absence from school an away from every one. This confirms that the IGT status represents a developmental risk factor in pediatric patients with CF. Furthermore, in this case it is conceivable that insulin glargine therapy has contributed preserving him from further clinical worsening, as we had observed in the previous years of COVID-19 pandemic.

According to the latest ISPAD guidelines, insulin therapy is the standard medical treatment in case of CFRD while it could be considered a choice in the case of prediabetes in CF patients showing significant comorbidities, such as difficulty in maintaining weight, poor linear growth, frequent pulmonary exacerbations or worsening of lung function. However, it is still debated whether to initiate insulin in patients with CF and prediabetes. Few studies on the use of insulin in prediabetic patients have been performed, demonstrating both therapeutic efficacy of insulin treatment [14, 26, 27] and not [9]. However, the latter is a study including both adult and pediatric subjects. Our clinical case underlines the potential role of insulin in the early stages of glucose derangements, even before the diagnosis of CFRD, in a young patient with compromised clinical conditions. CFRD diagnosis was made 4 years after the initiation of insulin therapy when the worsening of glycemic control appeared. This was probably due to the association of several factors: poor therapeutic compliance, pulmonary exacerbation, excessive consumption of high-calorie foods and physical inactivity (due to the COVID-19 pandemic lockdown). It is reasonable to speculate that the early insulin therapy allowed to delay the onset of CFRD and to have a more indolent clinical course of the disease. However, no sufficient evidence is available to demonstrate our hypothesis. Regarding treatment strategies in patients with CF and prediabetes, further investigations with observational trials are needed to confirm the usefulness of early insulin therapy in the prediabetes stage. Actually, two large studies are in progress to answer this question: “Cystic Fibrosis - Insulin Deficiency, Early Action” (ClinicalTrials.gov Identifier: NCT01100892) and “The Impact of Insulin Therapy on Protein Turnover in Pre-Diabetic Cystic Fibrosis Patients” (ClinicalTrials.gov Identifier: NCT02496780). The results of these studies will clarify if treatment of prediabetes in patients with CF is effective to delay the clinical decay of the underlying disease.

During the quarantine, diseases other than COVID-19 remained in the background and patients delayed their regular follow-up visits or did not worry about their symptoms aggravating the onset of many diseases as diabetic ketoacidosis. Blood glucose should be carefully monitored in diabetic patients with COVID19 infection as an increased risk of ketoacidosis has been reported in these patients [28]. Even among patients hospitalized for COVID-19 and without a history of diabetes, an increased mortality rate and prolonged hospitalization were reported in those whose blood glucose levels had increased during the hospitalization period. These data suggest that keeping blood glucose within normal limits can reduce complications and mortality [29]. In pediatric patients, comorbidities such as diabetes, chronic lung disease, tumors, immunodeficiencies, chronic renal failure, and neurological disorders may increase the morbidity-mortality risk related to COVID-19. These patients not only risk a more severe course of COVID19 infection, but also mismanagement of the underlying disease [30]. To reduce this risk, specific follow-up paths should be ensured for these patients even during times of a pandemic. It is essential to avoid the interruption of care for chronic patients even by using alternative pathways such as telemedicine when regular follow-up cannot be guaranteed.

## Data Availability

All data generated during this study are included in this published article and its supplementary information files.

## Abbreviations

ADA: American Diabetes Association
CF: Cystic Fibrosis
CFRD-FH-: Cystic Fibrosis-Related Diabetes less Fasting Hyperglycemia
CFRD-FH+: Cystic Fibrosis-Related Diabetes plus Fasting Hyperglycemia
CFRD: Cystic Fibrosis-Related Diabetes
CFTR: Cystic Fibrosis Transmembrane Conductance Regulator protein
FEV1: forced expiratory rate in the first second
FPG: Fasting Plasma Glucose
HbA1c: glycosylated haemoglobin
IGT: Impaired Glucose Tolerance
INDET: Indeterminate Glucose Tolerance
ISPAD: International Society for Pediatric and Adolescent Diabetes
NGT: Normal Glucose Tolerance
OGTT: Oral Glucose Tolerance Test
ppFEV1%: percent predicted forced expiratory rate in the first second
T1DM: Type 1 Diabetes Mellitus
T2DM: Type 2 Diabetes Mellitus
